# Spatiotemporal Distribution Characteristics and Driving Forces of PM2.5 in Three Urban Agglomerations of the Yangtze River Economic Belt

**DOI:** 10.3390/ijerph18052222

**Published:** 2021-02-24

**Authors:** Jin-Wei Yan, Fei Tao, Shuai-Qian Zhang, Shuang Lin, Tong Zhou

**Affiliations:** 1 School of Geographical Sciences, Nantong University, Nantong 226007, China; 1822021022@stmail.ntu.edu.cn (J.-W.Y.); 1921110012@stmail.ntu.edu.cn (S.-Q.Z.); 1921110003@stmail.ntu.edu.cn (S.L.); 2 Department of Geography, University of Wisconsin-Madison, Madison, WI 53706, USA; 3 Key Laboratory of Virtual Geographical Environment, MOE, Nanjing Normal University, Nanjing 210046, China

**Keywords:** urban agglomeration, PM2.5 concentrations, spatiotemporal distribution, geodetector, geographically weighted regression

## Abstract

As part of one of the five major national development strategies, the Yangtze River Economic Belt (YREB), including the three national-level urban agglomerations (the Cheng-Yu urban agglomeration (CY-UA), the Yangtze River Middle-Reach urban agglomeration (YRMR-UA), and the Yangtze River Delta urban agglomeration (YRD-UA)), plays an important role in China’s urban development and economic construction. However, the rapid economic growth of the past decades has caused frequent regional air pollution incidents, as indicated by high levels of fine particulate matter (PM2.5). Therefore, a driving force factor analysis based on the PM2.5 of the whole area would provide more information. This paper focuses on the three urban agglomerations in the YREB and uses exploratory data analysis and geostatistics methods to describe the spatiotemporal distribution patterns of air quality based on long-term PM2.5 series data from 2015 to 2018. First, the main driving factor of the spatial stratified heterogeneity of PM2.5 was determined through the Geodetector model, and then the influence mechanism of the factors with strong explanatory power was extrapolated using the Multiscale Geographically Weighted Regression (MGWR) models. The results showed that the number of enterprises, social public vehicles, total precipitation, wind speed, and green coverage in the built-up area had the most significant impacts on the distribution of PM2.5. The regression by MGWR was found to be more efficient than that by traditional Geographically Weighted Regression (GWR), further showing that the main factors varied significantly among the three urban agglomerations in affecting the special and temporal features.

## 1. Introduction

Fine particulate matter (PM2.5) is a type of particulate matter with an aerodynamic diameter of less than 2.5 μm. As the main source of air pollutants in urban areas [1], PM2.5 has taken a serious toll on human health. According to the report of the World Health Organization, 4.2 million people die from exposure to ambient air pollution every year worldwide [2]. Existing research showed that long-term exposure to high concentrations of PM2.5 might be accountable for at least 64 serious diseases [3,4,5] and that there are definite positive correlations between the concentration distribution of PM2.5 and the fatality rates of certain diseases [6,7].

China is experiencing rapid urbanization and has achieved a high degree of urban integration in the past two decades [8]. Several regions in the mainland of China have developed into national-level urban agglomerations and become important carriers of economic development [9]. However, this rapid development of cities is accompanied by serious burdens of air pollution and public health [10]. Comparative studies have shown that air pollution emissions on an urban agglomeration scale are more serious than those on an urban scale [11]. Therefore, studies on the spatiotemporal distribution characteristics of PM2.5 concentrations at the urban agglomeration level and an understanding of the driving factors will provide significant information to help improve urban air quality, reduce the health risk of residents, and promote the sustainable development of urban agglomeration.

At present, studies on PM2.5 mainly rely on data acquisition and assessment of the spatiotemporal distribution characteristics and driving factors. Data sources are mainly derived from environmental monitoring sites [12] and aerosol optical depth (AOD) production from remote sensing [13,14,15,16,17]. However, different data collection methods have also led to obvious discrepancies between the two data sets. The advantage of site statistics is its quick acquisition of high-precision, continuous-time, and temporal fine-grained PM2.5 data. Because of the discrete distribution of PM2.5 in the city built-up areas of the sites, site statistics cannot show the continuous spatial distributions of PM2.5 concentrations. Remote sensing technology can reflect spatial distribution characteristics of PM2.5, but accuracy is often affected by several factors, such as meteorological conditions and the collection sensors of the satellites. It is also difficult to obtain a continuous data set in a specific period, and the original image data also need a series of data preprocessing operations, such as atmospheric calibration and radiation correction, which is not a user-friendly process for non-professionals.

Research on the concentration distribution of PM2.5 has been conducted mainly from spatial and temporal perspectives. In the time dimension, according to statistical analyses of data collected on the trends of PM2.5 concentrations, such as the daily peak moments of PM2.5 in several different regions, conclusions such as the monthly mean PM2.5 typically exhibited a characteristic “U” shape in a given year—i.e., “low in summer and autumn but high in spring and winter in a yearly view” [18,19,20,21]. Other studies have paid attention to the regression predictions of atmospheric pollutant concentration values at different time granularities through machine learning models, such as the Back Propagation (BP) neural network [22], random forest model [23], hybrid neural network [24], and Long Short-Term Memory (LSTM) [25]. However, these studies only aimed at mining the statistical significance of the data itself. They ignored the description of the spatial distribution patterns of atmospheric pollutants and did not discuss the driving factors of PM2.5 production and changes. In the spatial dimension, models have been built and used to analyze the spatial distribution of air pollutants based on the Land-use Regression model (LUR) and the Geographically Weighted Regression (GWR) model [26,27,28]. Recent advancements in spatial robustness research have led to rapid developments in geostatistical models, such as the GWR [29,30] and LUR [31] models based on improved strategies. Further, models such as PCA-GWR, Multiscale Geographically Weighted Regression (MGWR), Geographically and Temporally Weighted (GTWR), and the geostatistical model combined with machine learning have produced more reliable regression results at spatiotemporal scales [32,33,34,35]. Due to the differences in their data sources, study areas, and influencing factor selection, however, such models can result in inconsistent conclusions. Studies at different spatial scales will also affect the model results. The large number of experiments studying the driving factors of PM2.5 have proven that socio-economic factors and natural factors are the main features that affect PM2.5 concentration level and spatial distribution [36]. Therefore, determining how to screen the specific indicators of the two influential factors has become particularly important, and researchers have been taking different approaches, such as using the geographic detector model [4,37,38], principal component analysis [29], and the correlation coefficient [39], to solve these problems. In the analysis of PM2.5 driving force factors, statistical models based on GWR [1] and the Spatial Durbin Model (SDM) [40] still play an important role. Li and Xu used the wavelet model [41] and the method of constructing multi-scale buffer zones [36], respectively, to explore the influence of various factors on air pollutants. However, the above studies often used a single model in the screening process of PM2.5 impact factors. In this paper, through the comprehensive use of various models and methods, we gradually analyzed the main impact factors of PM2.5 in the three major urban agglomerations of the Yangtze River Economic Belt, and improved the universality of the conclusions.

With the increasing incidents of haze in China, calls for integrated atmospheric management have been frequently made and implemented when regional air pollution incidents or severe air quality events occur. However, except for heavily polluted areas such as Beijing, Tianjin, and Hebei, studies on the regional integration of air quality management of factors affecting PM2.5 in other parts of China have not yet been conducted on a comprehensive large-scale [42]. The Cheng-Yu urban agglomeration (CY-UA), the Yangtze River Middle-Reach urban agglomeration (YRMR-UA), and the Yangtze River Delta urban agglomeration (YRD-UA) are three national city groups in the Yangtze River Economic Belt (YREB) that have an irreplaceable economic status in China. However, the long-term extensive mode of economic growth in these areas has also yielded a large-scale discharge of major air pollutants in the YREB. Although air quality has been improved in recent years, the pressure of smog control is still present [43]. Most of the existing studies focus on analysis within a certain urban agglomeration [37,44,45], while those covering the entire YREB suffer from a short period of analysis [46] and single model selection [37,43]; this results in poor interpretation ability of PM2.5 pollution in a long time series and a large space in the Yangtze River Economic Belt. Therefore, a comprehensive study of PM2.5 based on a long-term multi-spatial scale, multi-impact index, and multi-analysis model of the YREB is needed.

As discussed above, using a single perspective approach to study the spatiotemporal properties of air pollutants cannot accurately and comprehensively describe the patterns of changes in regional PM2.5 or the spatiotemporal distribution of PM2.5 and its driving forces. In addition, limited by the cost of data acquisition and the choice of research methods, large-scale PM2.5 studies are often influenced by the geographical environment, government policies, and other factors. It is thus difficult to reach a unified conclusion regarding the selection of factors affecting PM2.5. The present study employed the following approaches:

1. Data from 375 national environmental monitoring stations in the three major urban agglomerations of the YREB from 2015 to 2018 was used, from which the spatial autocorrelation of PM2.5 was summarized based on the perspective of urban agglomeration, and the spatial distribution patterns of PM2.5 and variation rule of PM2.5 concentrations at different time scales were described on a macro scale.

2. The main driving factors affecting the spatiotemporal differentiation of PM2.5 at a large spatial scale were explored by combining the above data with meteorological and socio-economic data of the same period and by using the exploratory data method and geographical detector model.

3. GWR and MGWR models were constructed based on the five strong explanatory power factors screened out to describe the driving force factors of the overall PM2.5 spatial distribution in the three urban agglomerations, and the change patterns of the most important influencing factors were analyzed in each urban agglomeration.

## 2. Materials and Methods

### 2.1. Study Area

As the longest river in China, the Yangtze River is located between 90°33′~122°25′ eastern longitude and 24°30′~35°45′ northern latitude, traversing central China from west to east. This river has played an extremely important role in promoting urban economic development within the basin and is known as China’s golden waterway. The YREB is formed along the river, spanning the three major regions of Eastern, Central, and Western China. This area covers 11 provinces and cities including Shanghai, Jiangsu, Zhejiang, Anhui, Jiangxi, Hubei, Hunan, Chongqing, Sichuan, Guizhou, and Yunnan. Since the release of the outline of Yangtze River Economic Belt development plan in September 2016, the YREB has formed a new development pattern of “one axis, two wings, three poles, and multiple points”. We take the “three poles” in the YREB (namely, Cheng-Yu urban agglomeration, Yangtze River Middle-Reach urban agglomeration, and Yangtze River Delta urban agglomeration) as our study area and analyze the spatiotemporal distribution characteristics and variation trends of PM2.5 and the driving factors of air pollution within the research scope by combining meteorological, socio-economic, and demographic data.

**Figure 1:**
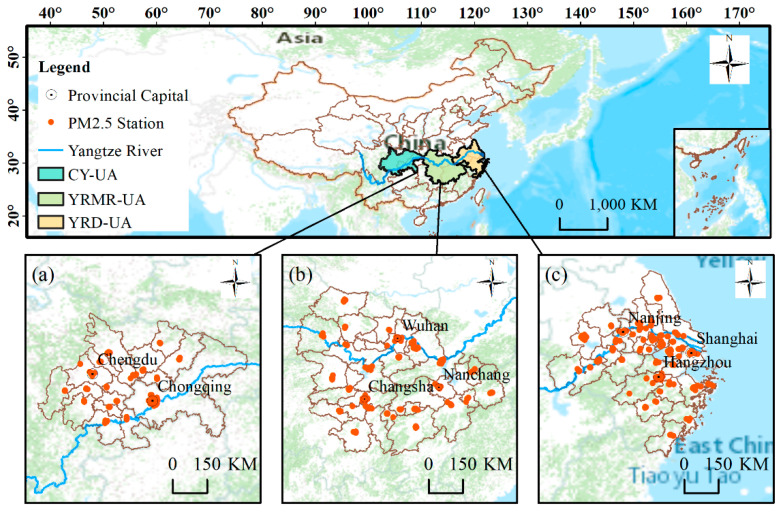
Location of the study area and spatial distribution of fine particulate matter (PM2.5) monitoring sites: (**a**) Cheng-Yu urban agglomeration (CY-UA), (**b**) Yangtze River Middle-Reach urban agglomeration (YRMR-UA), and (**c**) Yangtze River Delta urban agglomeration (YRD-UA).

The three major urban agglomerations of the YREB cover nine provinces and cities, including Shanghai, Jiangsu, Zhejiang, Anhui, Hubei, Hunan, Jiangxi, Chongqing, and Sichuan. The Cheng-Yu urban agglomeration with Chongqing and Chengdu as the center includes 17 cities in total and is an important platform for the western development strategy. The study area of Chongqing, Mianyang, Dazhou, and Ya’an does not cover the whole city. However, due to the difficulty in obtaining data from some districts, we selected the whole city data of Chongqing, Mianyang, Dazhou, and Ya’an for calculations (the same below). Wuhan is the center of the urban agglomeration in the middle reaches of the Yangtze River, which includes 31 cities in Hunan, Hubei, and Jiangxi provinces. Due to the lack of data on county-level cities directly under Xiantao, Qianjiang, and Tianmen, we do not study these cities. Shanghai is the core city of the YRD-UA, which includes 27 cities in the three provinces and one municipality including Jiangsu, Zhejiang, Anhui, and Shanghai, covering an area of 225,000 square kilometers. Meanwhile, we selected data from 375 research stations. Therefore, the newly added air quality monitoring stations were not used in this study. The geographical locations of the three urban agglomerations and the distribution of their internal air quality monitoring stations are shown in Figure 1.

### 2.2. Data Source and Description

The monthly, quarterly, and annual PM2.5 data were obtained from the China National Environmental Monitoring Station (http://www.cnemc.cn, last accessed on 15 July 2020). Hourly data from 375 air quality monitoring stations in the study area were used, and data preprocessing was conducted according to the requirements of the Environmental Air Quality Standard (GB3095-2012). Then, the monthly, and annual mean concentrations of each city were calculated based on hourly and daily data. Meteorological data were obtained from the China meteorological data network (http://data.cma.cn/market/index.html, last accessed on 20 July 2020) and the Hui-Ju data network (http://hz.zc12369.com/home/meteorologicalData/dataDetailsThreeYear/, last accessed on 21 July 2020). The hourly mean data of each city for 48 months were used in total, and the monthly mean meteorological data of each city were also obtained through mathematical calculation. Data on economics, traffic, city attributes, and education are collected from the National Bureau of Statistics released by the China statistical yearbook (http://www.stats.gov.cn/tjsj/ndsj/, last accessed on 18 July 2020). Other data are obtained from various statistical yearbooks. Population density and green coverage in the built-up area were obtained from the National Bureau of Statistics (http://www.stats.gov.cn/zjtj/gjtjj/201311/t20131108_457871.html, last accessed on 18 July 2020) released by the China city statistical yearbook. The types of data used in the study and their applications are outlined in Table 1.

In Table 1, the sampling interval of economy, traffic, urban attributes, education and other data is yearly. The sampling interval of meteorological data and PM2.5 data is hourly. According to the experimental requirements, different time scales are obtained through mathematical calculations.

### 2.3. Methodology

#### 2.3.1. Global and Local Spatial Autocorrelation Analysis

Tobler’s first law of geography states that objects are not distributed independently in space but are always correlated. To quantitatively analyze the spatial autocorrelation of air pollutants between adjacent areas, the Global Moran’s Index (Moran’s I) was calculated for PM2.5 [47]. The calculation formula of Global Moran’s *I* is as follows:

(1) $$I=\frac{n\sum_{i=1}^{n}\sum_{j=1}^{n}W_{ij}\left(x_{i}-\bar{x}\right)\left(x_{j}-\bar{x}\right)}{\sum_{i=1}^{n}{\left(x_{i}-\bar{x}\right)}^{2}\left(\sum_{i=1}^{n}\sum_{j=1}^{n}W_{ij}\right)},\left(i\neq j\right)$$

where *n* is the number of research units—in this study, the number of cities in the research sample area; $x_{i}$ and $x_{j}$ are the observed PM2.5 values of cities i and j, respectively; and $W_{ij}$ is a custom space weight matrix. If city i is adjacent to city j, $W_{ij}$ will be recorded as 1, otherwise it will be recorded as 0, thus the multidimensional spatial matrix is established. The value range of Moran I is [−1, 1]. When *I* is greater than 0, there is a positive spatial autocorrelation, and the larger the value is, the stronger the autocorrelation is. When *I* < 0, the space is negatively correlated. Meanwhile, a significance test of the *Z* value of Moran’s *I* value of the above results was carried out as follows:

(2) $$Z\left(I\right)=\frac{1-E\left(I\right)}{\sqrt{Var\left(I\right)}}$$

where E(I)=−1/(n−1), and Var(I) is the variance of I. At a 95% confidence level, $Z_{I}>1.96$ means that the autocorrelation of the positive space is significant, and $Z_{I}<-1.96$ means that PM2.5 has significant negative spatial autocorrelation. This indicates that the spatial autocorrelation is not obvious. When $-1.96<Z_{I}<1.96$, the spatial autocorrelation is not significant.

Because the global Moran’s I can only analyze the spatial autocorrelation of air pollutants from a global perspective, it cannot accurately find the spatial outliers [20]. Local autocorrelation analysis reflects the spatial agglomeration degree of similar attribute values around a region. The calculation formula for the local Moran’s *I* is as follows:

(3) $$I=\frac{n\left(x_{i}-\bar{x}\right)\sum_{j=1}^{m}W_{ij}\left(x_{j}-\bar{x}\right)}{\sum_{i=1}^{n}{\left(x_{i}-\bar{x}\right)}^{2}},\left(i\neq j\right)$$

where *n* is the number of cities; *m* is the number of cities adjacent to city *i* in geographical space; $x_{i}$ and $x_{j}$ are the PM2.5 concentration value of city i and j, respectively; and $W_{ij}$ is a custom space weight matrix. The local Moran’s *I* can also be tested using a standardized statistic *Z*. In local autocorrelation, at a 95% confidence level, |Z|>1.96 is significant, and |Z|>2.58 is extremely significant. At the same time, the local Moran can obtain four distribution modes: high–high correlation (H–H), low–low correlation (L–L), high–low correlation (H–L), and low–high correlation (L–H). If the PM2.5 concentration of city *i* and its surrounding cities is higher than the mean values of region, it presents an H–H correlation distribution, which is called a “hot zone”. If the PM2.5 concentration of city *i* and surrounding cities is lower than the regional mean, it presents an L–L correlation distribution, which is called a “cold zone”. When Z<1.96, if the concentration of PM2.5 in the city *i* is greater than or less than that in the surrounding city *j*, the concentration can be divided into two spatial distribution modes: the H–L type and the L–H type.

#### 2.3.2. Geodetector

Spatial stratification and heterogeneity are spatial manifestations of natural and socio-economic processes. Geodetector is an advanced statistical method proposed by the Institute of Geographic Sciences and Natural Resources Research under the Chinese Academy of Sciences, which is used to detect stratified heterogeneity in space and reveal the driving factors behind that heterogeneity [48]. In this paper, based on the meteorological and socio-economic data, we use the geodetector to screen and extract the most important driving factors of PM2.5 distribution in the YREB. The principle of the geographic detector is to divide the study area into several sub-regions. If the sum of the variances of each sub-region is less than the total variance of this region, there are spatial differentiation characteristics in the region. The geodetector can effectively identify the influence of factor variables on the spatial distribution of the results. In this paper, the Geodetector is used to calculate the explanatory power of socio-economic, population, and traffic factors and meteorological factors on the spatial differentiation of PM2.5 in the three urban agglomerations of the YREB. The calculation formula is as follows [49]:

(4) $$q=1-\frac{\sum_{h=1}^{L}N_{h}\sigma_{h}^{2}}{N\sigma^{2}}.$$

where *q* represents the explanatory power of a probe factor on PM2.5 spatial differentiation. The influencing factors are screened according to the *q* value. *h* is a count variable. *h* = 1, 2, 3, …, *L* represents the number of layers of the detection factor; *N* is the number of cities; $N_{h}$ represents the number of cities in layer h of the detection factor; $\sigma^{2}$ represents the variance of PM2.5 in three urban agglomerations; and $\sigma_{h}$ represents the variance of PM2.5 in layer *h*. The value range of *q* is [0, 1], where the larger the value of *q* is, the stronger the explanatory power of this factor for PM2.5 differentiation will be. Since the study area is bounded by the administrative region boundary, ArcGIS 10.2.2 is used to convert the study area into 10 × 10 km grid points, and the data information was extracted as input. Since geographical detectors are good at analyzing type quantities, discrete processing is required for continuous data [50]. In this paper, the natural discontinuous method in the ArcGIS10.2.2 software was used to divide the 11 detection factors collected into 8 categories. Meanwhile, the three submodels of Geodetector, namely factor detector, ecological detector and interaction detector, are used to explain the influence of driving force factors on the PM2.5 distribution. Factor detector was used to calculate the explanatory power of each impact factor on the spatial differentiation of PM2.5, which could quantitatively rank and screen the importance of PM2.5 driving factors. The ecological detector was used to compare if there is a significant difference in the influence of the two factors on the spatial distribution of PM2.5, which can also find out the important driving factors affecting PM2.5 in the YREB. The Interaction Detector evaluates whether the combined effects of two factors can increase or decrease the explanatory power of the dependent variable, or whether the effects of these factors on the spatial distribution of attributes are independent.

#### 2.3.3. GWR and MGWR Model

When analyzing the driving forces of PM2.5 distribution, it is necessary to fully consider the spatial non-stationarity caused by changes in the geographical factors [51]. Traditional regression methods, such as ordinary least squares (OLS), are global statistics that assume the studied relationships are constant over space. However, the GWR proposed by Brunsdon and Fotheringham can accurately predict the relationship between the dependent variables and the predictive variables of each location by constructing a local regression equation and considering the local heterogeneity of the regression coefficient in geographical space [52]. Therefore, we use this model to regress the spatial heterogeneity of PM2.5. The formula is as follows:

(5) $$y_{i}=\beta_{0}\left(\mu_{i},\gamma_{j}\right)+\beta_{1}\left(\mu_{i},\gamma_{j}\right)x_{i1}+\beta_{2}\left(\mu_{i},\gamma_{j}\right)x_{i2}+\cdots +\beta_{k}\left(\mu_{i},\gamma_{j}\right)x_{ik}+\varepsilon_{i}$$

where $y_{i}$ represents the explanatory value of the dependent variable of city *i*, which is the PM2.5 concentration of the city in this study; $\left(\mu_{i},\gamma_{i}\right)$ represents the geographic coordinates of city i; and $x_{i}$ represents the independent variable explanatory value of city *i*. In this paper, there is an economic factor, a trafficking factor, an urban attribute factor, and two meteorological factors; $\beta_{k}\left(\mu_{i},\gamma_{i}\right)$ is the regression parameter at the center of mass of city *i*. *ε_i_* represents a random error term. Here, the Gauss function is selected as the weight function, whose expression is as follows [52]:

(6) $$W_{ij}=\exp\left(-{\left(\frac{d_{ij}}{b}\right)}^{2}\right)$$

where $W_{ij}$ represents the weight influence between city *i* and city *j*, and *b* represents the bandwidth. The larger the bandwidth *b* is, the slower the weight influence decreases with an increase in distance $d_{ij}$; the smaller the bandwidth *b* is, the faster the weight influence decreases with an increase of distance $d_{ij}$. In this paper, the AICc guidelines are used to optimize bandwidth.

Although GWR considers the influence of local regression in the spatial analysis compared with other statistical methods, it still sets all the relationships involved as constant at a spatial scale and does not allow the analysis of the relationships of geographical phenomena at different scales [33]. However, this constant analysis at a spatial scale is not reasonable to study air quality. Therefore, this paper further uses the MGWR model to analyze the spatial heterogeneity of PM2.5, and discusses the change process of the main influencing factors on PM2.5 in the three major urban agglomerations of the YREB based on the experimental results.

MGWR is an extension of GWR that allows one to study relationships at varying spatial scales and achieves this goal by using a varying bandwidth as opposed to single, constant bandwidth for the entire study area [53]. MGWR can be formulated as:

(7) $$y_{i}=\sum_{j=1}^{n}a_{j}x_{ij}+\sum_{j=n+1}^{m}\beta_{j}\left(\mu_{i},\gamma_{i}\right)x_{ij}+\varepsilon_{i}$$

where $\left(\mu_{i},\gamma_{j}\right)$ represents the geographical coordinates of i city, j represents the number of cities, $x_{i}$ represents the independent variable explanatory value of city *i*, *X_ij_* represents the observation of the *j* independent variable at location *i*; $a_{j}$ is the regression coefficient of the global variable, $\beta_{j}$ is the regression coefficient of the local variables, and $\varepsilon_{i}$ represents a random error term. Similarly, the Gauss function is selected as the weight function in MGWR. The specific introduction is the same as above. We used the MGWR2.2 software which was presented by the School of Geography and Urban Planning, Arizona State University to undertake all calibrations (https://sgsup.asu.edu/sparc/mgwr last accessed on 18 July 2020).

## 3. Results

### 3.1. Spatial and Temporal Distribution of PM2.5

#### 3.1.1. Statistical Analysis

**Figure 2:**
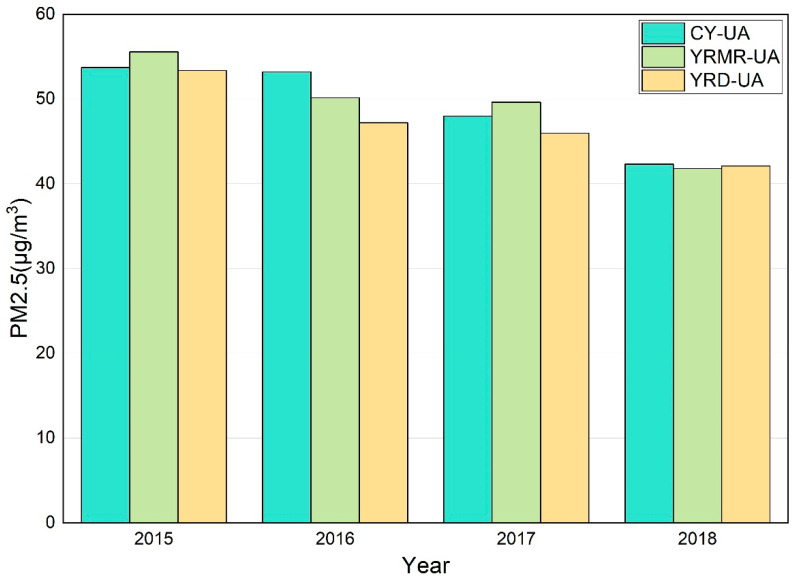
Annual PM2.5 concentrations averaged over the three urban agglomerations in subsequent years.

Compared with cities, the contradiction between economic development and environmental protection in urban agglomerations is more prominent, and the problem of air pollution is more complicated [19]. Therefore, it is necessary to conduct a statistical analysis of the changes in PM2.5 in the three urban agglomerations over four years. This paper uses hourly PM2.5 statistics from monitoring stations in each city to calculate the overall annual average changes in the urban agglomeration. Year by year, the PM2.5 total concentration in the three urban agglomerations showed a trend of decline during the past four years, with a cumulative decrease of more than $10\;\mu g/m^{3}$(Figure 2). The annual mean concentration of PM2.5 is sufficient to meet the second level defined by Chinese standards $\left(35\;\mu g/m^{3}<\mathrm{PM}2.5<75\;\mu g/m^{3}\right)$ but falls to the third tier under the stricter American standards for sensitive groups $\left(35\;\mu g/m^{3}<\mathrm{PM}2.5<55\;\mu g/m^{3}\right)$; thus, the air quality situation needs to be further improved.

Through comparison, we analyzed the changing trend of PM2.5 concentration in three urban agglomerations. The declining trend of CY-UA was divided into two stages before and after 2016. The PM2.5 concentration of YRMR-UA decreased the most significantly, and the decreasing trend of PM2.5 concentrations in YRD-UA was relatively stable over the four years. Notably, since 2016, the PM2.5 concentration of CY-UA has decreased significantly overall; the total PM2.5 concentration of the YRMR-UA also dropped significantly in 2018. The phenomenon of PM2.5 concentration decline is related to the time and place of the two speeches of General Secretary Xi Jinping at the symposium on promoting YREB development (the first is Chongqing in 2016 and the second is Wuhan in 2018). This speech noted that the restoration of the ecological environment in the Yangtze River should be overwhelmingly prioritized. At the same time, the Ten Atmospheric Policies, Outline of YREB Development Plan, Plan for the Ecological and Environmental Protection of the Yangtze Economic Belt, and Working Memorandum on Strengthening Cooperation between Sichuan Province and Chongqing Province in Building Cheng-Yu Urban Agglomeration all indicate that the environmental policies formulated by national or local government departments play a guiding role in improving air quality.

**Figure 3:**
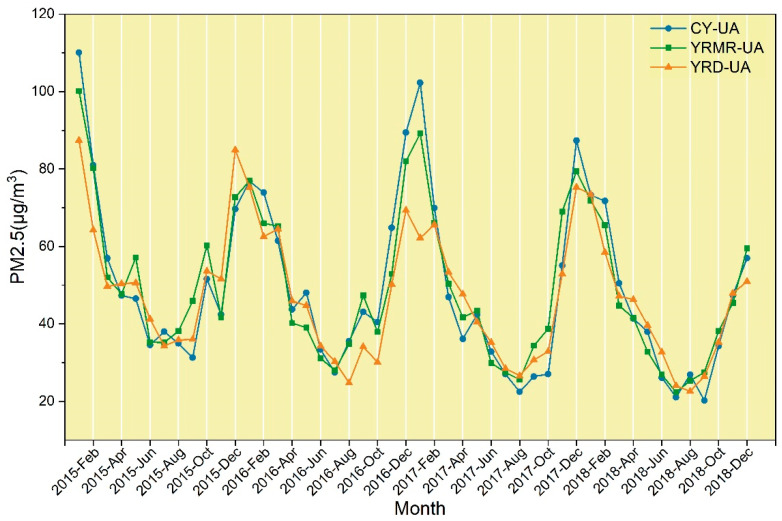
Statistical chart of the monthly mean PM2.5 concentration in three urban agglomerations.

After annual PM2.5 concentrations analysis, we describe the monthly variation of PM2.5. Figure 3 shows the monthly change trend of PM2.5. It can be concluded from the figure that (1) the PM2.5 concentration shows a cyclical downward trend as a whole. (2) The fluctuation trends of PM2.5 concentrations in the three urban agglomerations are basically the same, Moreover, the temporal variation trend of PM2.5 over a year also conforms to the U-shaped variation law proposed by previous scholars [54]. This means that the air quality is better in summer and autumn, and the PM2.5 concentration is the lowest in autumn. This characteristic is also reflected by the fewer months in 2015 with good air quality $\left(\mathrm{PM}2.5<35\;\mu g/m^{3}\right)$ (June, August, and September in CY-UA and July in YRD-UA). In the following three years, the air quality in summer and autumn was mostly excellent with proper environmental governance, and the monthly average PM2.5 concentration also kept decreasing. (3) Although the overall trend indicates a decline, there are also abnormal phenomena showing rising concentrations. From the winter of 2016 to the spring of 2017, the PM2.5 concentrations of YRMR-UA and CY-UA were both higher than those during the same period in the previous year and the next year.

**Figure 4:**
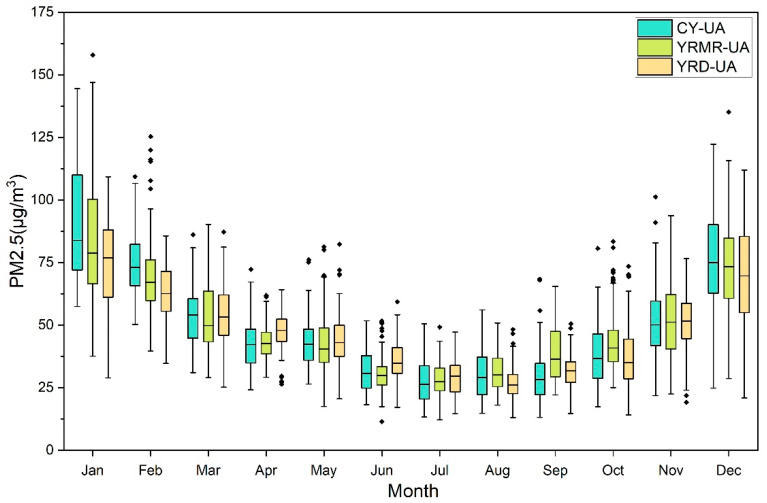
Monthly box diagram of PM2.5 concentrations in three urban agglomerations from 2015 to 2018. If there are obvious black spots on the top and bottom of the box graph, it corresponds to the description in the corresponding part in the section.

To further analyze the reasons for the emergence of this special situation, in Figure 4, a box chart of the monthly mean data of all cities in the three urban agglomerations from 2015 to 2018 is illustrated by month to capture the PM2.5 concentration anomaly in the urban agglomeration. In Figure 4, the YRMR-UA and CY-UA in December 2016 and January 2017 contain obvious high-pollution cities; the median and upper limits of the data as a whole were also much higher than the levels of the two years prior and following the same period. This explains the abnormal rise in PM2.5 concentrations for YRMR-UA and CY-UA from the winter of 2016 to the spring of 2017. According to the median line of each urban agglomeration in the box chart, we clearly confirmed that the PM2.5 concentration in a year showed a U-shaped pattern. The minimum concentrations of CY-UA and YRMR-UA were in July, while that of YRD-UA was delayed by one month. We found that the highest value of the median line of the box graph appeared in January of CY-UA, followed by January of YRMR-UA. This reflected that the pollution concentration of CY-UA and YRMR-UA changed greatly, while the pollution concentration of YRD-UA PM2.5 changed slightly.

The numerical dispersion of PM2.5 in three urban agglomerations over four years was analyzed month by month, and the following conclusions were obtained: (1) In the Cheng-Yu urban agglomeration, the PM2.5 concentration is evenly distributed in June, July, and August in summer and in December, January, and February in winter. The more severe outlier points in the remaining months are Neijiang in May 2015 and Zigong in October of the same year, Chongqing in September 2016, and Zigong and Chengdu in November 2016. Most of these cities are located in the east-central part of CY-UA. Relevant departments should pay more attention to this phenomenon and formulate targeted regional joint prevention and control policies for air pollution. (2) For YRD-UA, the time of the occurrence of PM2.5 concentration outliers is opposite to that of CY-UA—mostly near the bottom of the U-shaped curve. The median PM2.5 concentration in these months remained at a low level, and local PM2.5 pollution incidents led to a small number of urban outliers. (3) In the three urban agglomerations, the greatest number of outliers in PM2.5 concentration occurred in the YRMR-UA. A large number of extreme values were found for February, June, and October. This phenomenon indicates that the regional PM2.5 pollution during those months was relatively significant and that there were several places with high PM2.5 pollution values at the same time.

#### 3.1.2. PM2.5 Spatial Distribution

**Figure 5:**
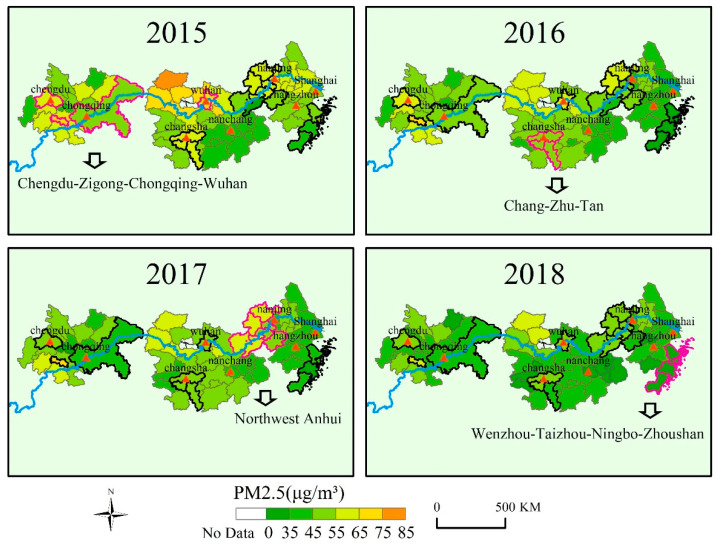
Spatial distribution of the annual mean PM2.5 in three urban agglomerations. The red triangle marks the locations of the provincial capital city and the municipality directly under the central government, and the others are marked with arrows.

This section mainly analyzes the spatial distribution characteristics of PM2.5 and its variation trends. Figure 5 shows the main characteristics of PM2.5 concentrations in the three urban agglomerations of the YERB from 2015 to 2018. The major observations were as follows: (1) The PM2.5 concentration in the provincial capitals and municipalities directly under the central government decreased more significantly than those in other prefecture-level cities. (2) PM2.5 pollution in the middle reaches of the Yangtze River is more serious than in the upper and lower reaches. PM2.5 pollution on the north bank of the Yangtze River is more severe than that on the south bank. (3) In the three urban agglomerations, PM2.5 is mainly distributed in five regions, but the causes are different. In the urban agglomeration of northwest Anhui and the Chang-Zhu-Tan, due to the economic development structure, the proportion of secondary industry is relatively high, which makes PM2.5 pollution centration higher However, the high PM2.5 distribution in the Wuhan metropolitan area and the Chengdu and Chongqing areas is closely related to economic activities and population density [41]. The terrain of Zigong area is low mountains and hills, which is located in the center of southern Sichuan airflow. In this area, the air mobility is poor, and pollutants can easily gather and become difficult to diffuse, resulting in a higher PM2.5 concentration than in the surrounding areas. (4) The distribution characteristics of PM2.5 also vary significantly between different provinces. The distribution of Wenzhou, Taizhou, Ningbo, and Zhoushan in Zhejiang province are mainly affected by natural factors such as weather and topography. The cities are located in coastal areas with more rainfall [55], so the air quality has remained at a good level for four years. In Jiangxi Province, the economic development in the northwest is better than that in the southeast, which leads to the difference in PM2.5 concentration distribution. Due to the adjustment of economic development structure, the improvement of green coverage in the built-up area, the increase of government spending on environmental protection, and good education foundation, Jiangsu has not experienced long-term and large-scale PM2.5 pollution during economic development [56].

**Figure 6:**
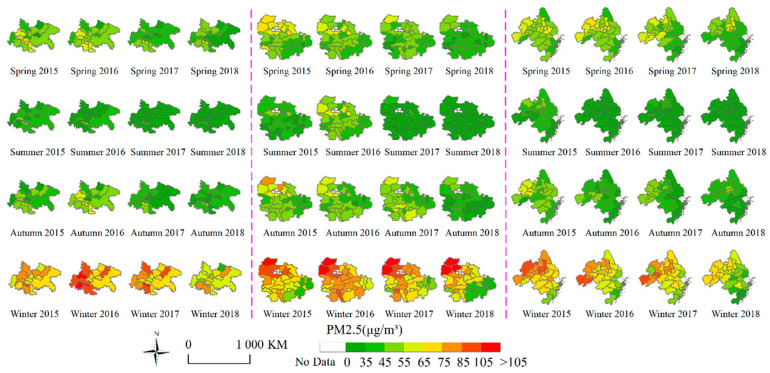
The seasonal distribution of PM2.5 from 2015 to 2018. From left to right are CY-UA, YRMR-UA, and YRD-UA.

By calculating the seasonal average value, the variation trend of PM2.5 in three urban agglomerations is deeply discussed. Each season is divided into three months: spring (March, April, and May), summer (June, July, and August), autumn (September, October, and November), and winter (December, January, and February of the following year). The spatiotemporal distribution of each season in four years is shown in Figure 6. Some conclusions can be drawn as follows. (1) The PM2.5 distribution in all agglomerations has a specific directionality, showing a diagonal distribution that is low in the southeast and high in the northwest, although this phenomenon only gradually manifested in CY-UA after 2017 and 2018. (2) From a seasonal perspective, it is found that the air quality of the three urban agglomerations was better in summer and autumn, and worse in spring and winter, and the air quality was improved year by year as a whole. (3) Finally, the seasonal variation of PM2.5 in each urban agglomeration was discussed on a time scale. In spring, the regions with high CY-UA pollution were concentrated in several cities in the central region, and high pollution decreased from north to south. The high pollution level of YRMR-UA decreased gradually from northwest Hubei province and central Hunan province to southeast Hubei Province. The PM2.5 pollution of the YRMR-UA was mainly distributed in Anhui province and northwest Jiangsu Province. In the four-year change process, there was a spatial process moving from west to east with high pollution. By 2018, areas with high PM2.5 pollution remained in Yangzhou, Taizhou, Zhenjiang, and Changzhou.

In summer, with Suining as the center, the CY-UA assumed a cross-shape and improved year by year; the distribution of PM2.5 in the YRMR-UA along both sides of the Yangtze River was also improved year by year. The PM2.5 concentration of YRD-UA was divided into north and south distribution with Xuancheng, Huzhou, and Jiaxing as boundary lines. In autumn, CY-UA and YRMR-UA experienced more random pollution in the four years, among which the PM2.5 concentration in Xiangyang, Yichang, and Jingmen in northwest Hubei province dropped the most obvious. Air quality in the northeast improved significantly in CY-UA during the fall of 2017. In 2015 and 2018, the PM2.5 concentration in the YRD-UA showed a spatial distribution trend of a gradual decrease from northwest to southeast and could be roughly divided into Zhejiang Province, with the best air quality according to administrative provinces; Jiangsu with medium air quality; and Anhui with the worst air quality. For 2016 and 2017, the PM2.5 concentration distribution in YRD-UA from east to west can be divided using Hangzhou, Huzhou, Suzhou, Nantong, and Yancheng as the boundary; individual cities did not affect the overall distribution.

In winter, PM2.5 pollution reached its peak, at which point its distribution became more diversified, and its classification in urban agglomerations became more complicated. The PM2.5 pollution of CY-UA was found to be the most serious among the three urban agglomerations. A large area of severe pollution occurred in the southwest in 2016 and continued into 2017, at which point the spatial distribution of Yibin in Mianyang was still the most polluted area. In 2015 and 2018, the most polluted areas were scattered. High and medium-concentrations of pollution in the YRMR-UA were mostly distributed in the three provinces with the most serious pollution areas in northern Hubei province. However, unlike the various characteristics in autumn, the air quality in the region did not show obvious improvement over the four years. With the Yangtze River as the boundary, the PM2.5 pollution in the south of Jiangxi province was significantly better than that in the north. The most seriously polluted area of the YRD-UA was dominated by Hefei in 2015 and distributed in Anhui and northern Jiangsu. By 2018, the most severely polluted area was reduced to only Anhui. Although the PM2.5 concentration in Anhui province is also gradually decreasing, its air pollution control remains a challenging task.

#### 3.1.3. PM2.5 Spatial Correlation Analysis

From the view of time series, the positive spatial correlation of YRD-UA became more and more obvious in four years, while definite laws did not be found of YRMR-UA and CY-UA at the same time. From the perspective of urban agglomerations, the spatial autocorrelation of PM2.5 in the three urban agglomerations was different, and the significance level was inversely proportional to the PM2.5 concentration value [40]. In the significance test of global Moran’s Index, the results of CY-UA were the worst, while more than half of the YRMR-UA passed the test, and almost all of YRD-UA reached 0.001, showing the correlation on a significant level.

The spatial autocorrelation of PM2.5 in YRD-UA was analyzed in Table 2. On the annual scale. In the case of |*Z*| > 1.96, YRD-UA’s Moran’s I index values are 0.285, 0.430, 0.573, and 0.610. These values indicate that the PM2.5 of YRD-UA tends to be more concentrated, which requires regional unified coordination and comprehensive treatment. At a seasonal scale, the spatial autocorrelation of PM2.5 decreased first and then increased. On a monthly scale, Moran’s I index values of YRD-UA are still high in January to March and October to December.

According to Table 3, the annual Moran’s I index value of YRMR-UA first decreased and then increased, indicating that the spatial autocorrelation of PM2.5 in this region first weakened and then increased. On the seasonal scale, the concentration trend of pollutants in spring and winter was more obvious, while *p* of summer didn’t pass the significance test. On the monthly scale, only the values of Moran’s I index of January, February, March and December are larger, showing a strong spatial autocorrelation of PM2.5.

This part focuses on analyzing the global Moran’s I for CY-UA. At an annual scale, CY-UA only passed the 0.001 significance test in 2017, and the Moran’s I index value was only 0.286 in the case of |Z|>1.96. At a seasonal scale, only summer passed the 0.05 significance test, indicating that the CY-UA spatial clustering characteristics were not significant. In the spring, summer, and autumn of 2017 and the summer season of all four years, the spatial differences in the PM2.5 concentration between CY-UA cities were small. On a monthly scale, the CY-UA values in March, April, and May were significant at a level of 0.05 and presented a certain positive spatial correlation; November presented a better spatial positive correlation at a 0.001 level of confidence, which is also verified the temporal characteristics of PM2.5. The significance tests for the remaining months are not discussed (Table 4).

**Figure 7:**
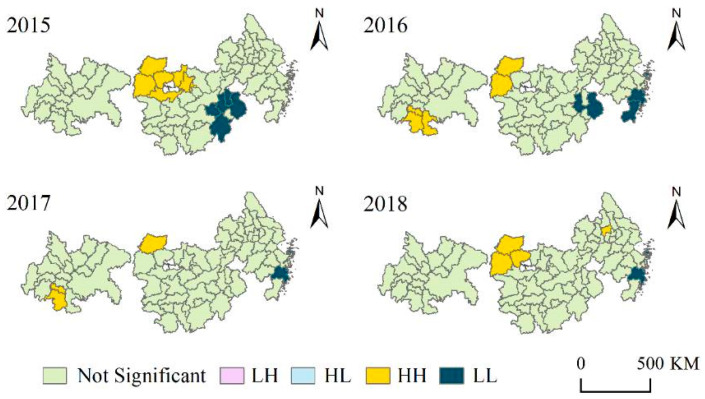
The results of the local Moran analysis and the spatiotemporal changes of the cluster pattern of “Low–Low” and “High–High” in the three urban agglomerations over the past four years.

Since global spatial autocorrelation cannot reflect regional differences, the local spatial correlation method was used to explore the local concentrations of PM2.5 in the three urban agglomerations. Figure 7 shows the positive correlation cluster patterns of “high–high” and “low–low” in the three urban agglomerations within four years, indicating obvious changes and shifts in spatial distribution. In 2015, the Wuhan metropolitan area showed an obviously high concentration of PM2.5, while a low concentration of PM2.5 occurred in Jiangxi province. By 2016, Xiangyang and Yichang were still among the places with high concentration values, and Luzhou, Yibin, and Zigong in the Cheng-Yu urban agglomeration also showed a high concentration. Shangrao of Jiangxi province remains a low-value gathering place, and YRD-UA also has two low-value gathering places: Zhoushan, Taizhou, and Wenzhou. The local spatial concentrations of PM2.5 in 2017 were roughly similar to those in 2016. By 2018, a new high concentration area was added to Zhenjiang, while the high concentration area of CY-UA disappeared.

Two typical concentrations of PM2.5 were analyzed. The Wuhan metropolitan area has a strong positive correlation in PM2.5 space, while Xiangyang has always appeared in the “high–high” aggregation model as a PM2.5 pollution center that affects the air quality of the surrounding cities. According to the time series analysis, the concentration of PM2.5 clustering in the Wuhan metropolitan area gradually decreased in the first three years, and the correlation between cities weakened, but this trend rebounded in 2018. Since 2016, Taizhou, Zhoushan, and other coastal cities have experienced low concentration levels, reflecting the strong positive correlation of PM2.5 at a lower level in the region. However, in terms of time series, such low-concentration PM2.5 clustering in Taizhou and Zhoushan was still present in 2017 and 2018, indicating more stable performance in the local correlation.

### 3.2. Analysis of Driving Force Factors of PM2.5

This section mainly discusses which factors have the strongest correlation with PM2.5 in the time series and which factors affect the spatial distribution of PM2.5. In previous studies, GDP, industrial production, built-up areas, use of energy, population density, and other factors were often selected as social influencing factors [37,57], while temperature, humidity, precipitation, and other meteorological conditions were selected as natural factors [58]. However, in most of these articles, the selection of driving factors was more dependent on the conclusions of other scholars than on experimental explanations. This strongly restricts the subsequent analysis results and may lead to inappropriate factors entering the analysis model. In the present study, the seven natural factors and eleven social factors that we collected were analyzed and screened step by step. In this way, we obtained a comprehensive selection of impact factors suitable for large-scale spatial research and providing scientific guidance for subsequent research.

#### 3.2.1. Analysis of Meteorological Factors

As a natural factor, meteorological conditions play an important role in the propagation of particulate matter and the concentration of atmospheric pollutants [29,59]. However, the present study found that the same meteorological factors are affected by objective factors, such as the different climatic zones, geographical locations, and spatial scopes of the study area, and had certain differences in their ability to influence PM2.5 in different regions. In this study, we used the meteorological data of the three major urban agglomerations over 48 months to explore the correlation between various meteorological factors and PM2.5 concentration.

**Figure 8:**
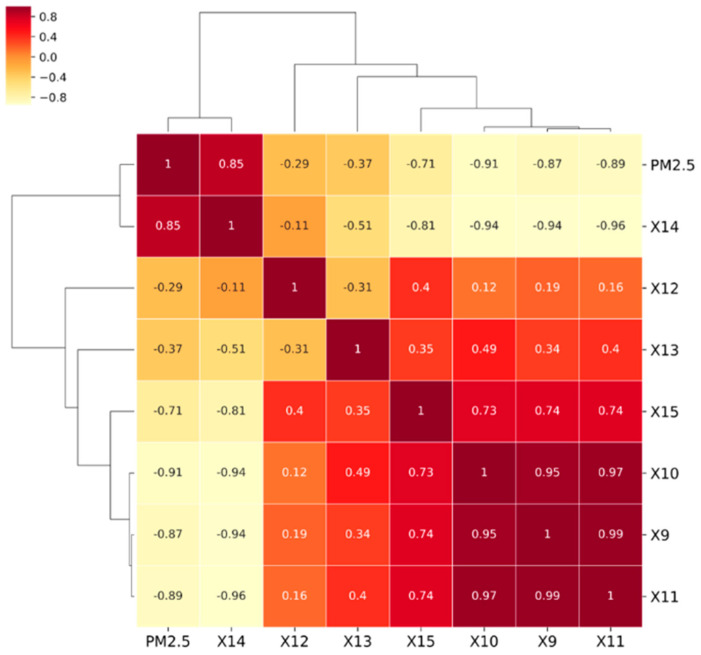
Cluster thermograph. The correlation between various meteorological factors and PM2.5 and the autocorrelation between the factors based on clustering.

Through the thermal diagram (Figure 8), we found that except for the positive correlation between air pressure and PM2.5, the other six indexes all showed a negative correlation with PM2.5 concentration. The correlation between temperature and PM2.5 concentration was the strongest, followed by that between pressure and precipitation. The influence of humidity and wind speed on PM2.5 was relatively weak. However, when the minimum distance clustering method was used for the hierarchical clustering of each factor, it was found that minimum temperature and average temperature could easily be grouped due to their strong data correlation, so they were removed in the subsequent analysis.

Qualitative cluster analysis showed that there was a certain correlation between meteorological factors. If the meteorological factors directly participated in the subsequent modeling process without screening, the GWR model could be affected by multicollinearity, producing errors or offering no results in the analysis results [60]. Therefore, the experiment further calculated the variance inflation factors (VIFs) of each meteorological factor to test the multicollinearity of the data [61]. Previous studies have pointed out that it was appropriate to set the VIF value between 2.5 and 10 [62], but the higher the VIF value, the stronger the collinearity between the influence factors. The above research has found that the high collinearity between the influence factors will have a certain impact on the GWR Model results. [63] In the results, we set the VIFs value to no more than 5 [64] for each factor. As shown in Table 5, only three meteorological factors—humidity, wind speed, and precipitation—passed the multicollinearity test.

#### 3.2.2. Analysis of Humanity Factors

We analyzed the spatial differentiation characteristics of PM2.5 influencing factors by adopting the three models in the geographic detector, and the following conclusions were drawn.

(1) Factor detector. The factor detector is mainly used to detect the explanatory power of influencing factors for some phenomena. In Table 6, which provides the Factor_detector analysis results, the influence of each factor on the spatial heterogeneity of PM2.5 passed the significance test. By comparison, in the three urban agglomerations of the YREB, the explanatory power of the spatial differentiation of the top five PM2.5 impact factors was ranked as follows: enterprises (X3) > total precipitation (X15) > social public vehicles (X5) > wind speed (X13) > green coverage in the built-up area (X7). These five factors were not completely attributed to socio-economic factors or meteorological factors, which indicates that the spatial heterogeneity of PM2.5 in the three urban agglomerations of the YREB is affected by multiple factors. Five factors with the strongest explanatory power, including enterprises, total precipitation, social public vehicles, wind speed, and green coverage in the built-up area were selected via the principal component analysis. These factors were able to explain the causes of PM2.5 spatial differentiation in nearly half of the three urban agglomerations, thereby simplifying the scientific parameters for large-scale spatial regression analysis.

(2) Ecological detector. Ecological detectors were used to compare whether there were significant differences in the influence of various influencing factors on the spatial distribution of PM2.5. In Table 7, Y represents the two factors that have significant differences in the spatial distribution of PM2.5, while N represents no significant difference. The results showed the following: (1) There are significant differences between the enterprises (X3) and other factors on the distribution of PM2.5 concentration in the three urban agglomerations. (2) In addition to the social public vehicles (X5), the total precipitation (X15) and the other nine factors also have significant differences in the spatial distribution of PM2.5. (3) In the remaining nine factors, except for social public vehicles (X5), population (X2), enterprises (X3), and humanity (X12), the other five factors had no significant effect on PM2.5 concentration distribution.

(3) Interaction detector. The Interaction_detector module can be used to compare the difference of the explanatory power of each factor superposed by two factors and that of a single factor. The analysis results in Table 8 show that the interaction of any two factors had a positive enhancement effect on the increase in the PM2.5 concentration and that this enhancement relationship varied between two times and ten times. This conclusion can be found by comparing the explanatory power of a single influence factor in Table 6. Especially when GDP (X1), population density (X6), the proportion of secondary industry (X2), and humidity (X12) were combined with other factors, their impact on PM2.5 increased the most. This discovery could facilitate the comprehensive treatment of air pollution. Meanwhile, when the enterprise (X3) interacts with other factors, its influence on the PM2.5 distribution difference of the three urban agglomerations is the strongest on the whole. Wind speed (X13) and total precipitation (X15) also appeared to exert a strong influence on the PM2.5 precipitation in the three major urban agglomerations when they interacted with most factors. This phenomenon indicates that meteorological factors as “catalysts” can enhance the explanatory power of other factors for PM2.5 concentration.

## 4. Discussion

We borrowed the idea of principal component analysis to select the first five factors with the strongest explanatory power, including enterprises, total precipitation, social vehicle ownership, wind speed, and green coverage, in the built-up area. These factors were able to explain the causes of PM2.5 spatial differentiation in nearly half of the three urban agglomerations, thereby simplifying the scientific parameters for large-scale spatial regression analysis.

**Figure 9:**
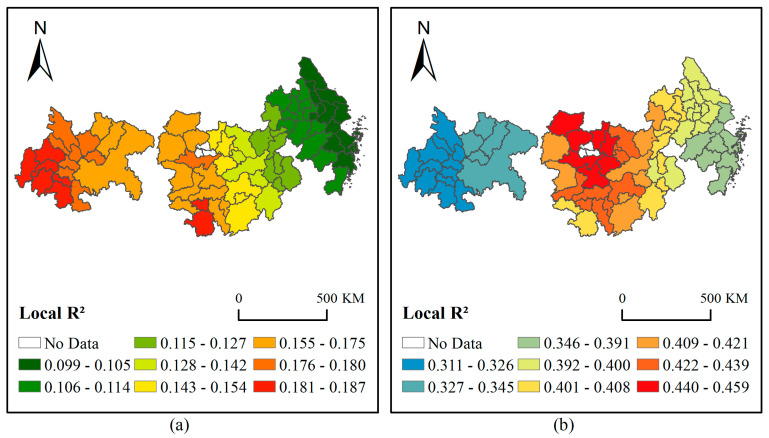
A comparison of the Geographically Weighted Regression (GWR) and Multiscale Geographically Weighted Regression (MGWR) local regression effects for 2018, with GWR on the left (**a**) and MGWR on the right (**b**).

Based on GWR and MGWR models, the above five indicators were selected for geographic regression analysis modeling for PM2.5 from 2015 to 2018. Figure 9 shows the modeling results for 2018. The main findings are as follows: (1) The spatial distribution patterns obtained by the two models were different. For the Local R² of GWR, YRD-UA was the smallest, and CY-UA was the largest. The local regression effect of MGWR was the worst for CY-UA and the best for YRMR-UA. The regression results indicated that different models had some differences in their feature extraction of explanatory factors. (2) From the regression results, the local regression coefficient of MGWR was significantly higher than that of GWR. Since MGWR allowed the optimization of covariate specific bandwidth rather than producing a single average bandwidth applicable to all relationships [52], making the model more sensitive to capturing details, the R² effect of MGWR was much better than that of GWR. (3) In Table 9, we list more regression outcome parameters for the GWR and MGWR models over four years. In addition to R², the residual squares of MGWR was much smaller than that of the GWR model, and the selection of parameter Sigma was also more rational. To sum up, MGWR in a large-scale spatial study was better than the GWR model in its overall regression effect, year-over-year stability, and local detailed description, making this model more suitable for multi-factor regression analysis from the perspective of urban agglomeration.

The bandwidth and local regression coefficients are commonly used for the impact analysis of driving force factors [60,65]. The most significant advantage of the MGWR model was that it not only allowed the spatial variation of each parameter estimate but also generated a separate optimal bandwidth for the conditional relationship between the response variable and each predictive variable, simulating the spatial variation process under different spatial scales. In Table 10, we outline these statistics, including the enterprises, total precipitation, social vehicle ownership, wind speed, and green coverage in the built-up area, as well as the effective number. The variables with large bandwidth will have a wide spatial influence on the dependent variable (PM2.5 concentration). In contrast, variables with a small bandwidth affected the dependent variable (PM2.5 concentration) at a local scale. Effective number of parameters (ENP) offered a compromise between the variance of the fitting value and the deviation of the coefficient estimate value, which was used to measure the value of the equilibrium point. In the first two years, green coverage in the built-up area (X7) had the minimum bandwidth. In the second two years, the factor of the minimum bandwidth and the maximum ENP value were both wind speed (X13). This reflected the change process of the influencing factors of spatial heterogeneity.

The calculated annual average of the local regression coefficients is shown in Table 11 and roughly reflects the changing trend of the most important factor affecting the PM2.5 of the urban agglomerations. The results showed the following: (1) In 2015, the most important influencing factors of the three urban agglomerations were all different—CY-UA: wind speed (X13); YRMR-UA: green coverage in the built-up area (X7); YRD-UA: total precipitation (X15). (2) Social public vehicles (X5), which were the most important influencing factor, were responsible for CY-UA and YRMR-UA, while the most important influencing factor for PM2.5 for the YRD-UA was wind speed (X13). (3) By 2017, the most important influencing factors of the three urban agglomerations changed again. The most important driving force of CY-UA changed from socio-economic factors to the meteorological factor of total precipitation (X15), while enterprises (X3)—as a socio-economic factor—influenced the PM2.5 concentration of the remaining two urban agglomerations. (4) In the analysis results of 2018, the most important impact factor of the three urban agglomerations was unified as wind speed (X13), indicating that meteorological factors played a dominant role in spatial regression modeling during this year.

In summary, from multiple spatial scales, this study objectively describes the spatial and temporal distribution of PM2.5 in the three urban agglomerations of the YREB from 2015 to 2018 and comprehensively uses a variety of analysis methods such as correlation analysis and geodetector. It significantly improves the applicability and universality of the analysis model and provides an effective method for subsequent studies. However, due to the limitation of time granularity of statistical yearbook data, GWR model and MGWR model only analyzed the interannual variation rule of PM2.5 driving factors, which is also the direction to be improved in subsequent experiments.

## 5. Conclusions

This paper took PM2.5 data from China’s environmental monitoring sites from 2015 to 2018 as its main research object and combined socioeconomic data and meteorological data from the same period to discuss the spatiotemporal distribution characteristics and the driving force factors of PM2.5 in the three urban agglomerations of the YREB. The spatial autocorrelation and spatial heterogeneity of PM2.5 from the perspective of urban agglomeration were analyzed, and the variations in this characteristic under different time granularities were presented using exploratory spatial analysis and a geographical statistical model. The main conclusions of the study are as follows: (1) Over the past four years, PM2.5 concentrations in the three urban agglomerations have obviously declined. In terms of specific agglomerations, the decrease of PM2.5 in the Yangtze River Middle-Reach urban agglomeration was the largest. Specifically, the PM2.5 concentration in the provincial capitals and municipalities has dropped the most. The analysis on the seasonal scale showed that the concentration of pollutants is high in spring and winter, while the concentration of PM2.5 is low in summer and autumn. (2) In terms of spatial distribution, PM2.5 pollution in the Yangtze River Middle-Reach urban agglomeration was more serious than that in the other two urban agglomerations, and that in the north bank of the Yangtze River was more serious than that in the south bank. Furthermore, the discussion of spatial autocorrelation shows the changing trend of high and low pollutant concentration areas. (3) Based on the correlation analysis and geographical detector model, we simplified the initial eleven impact factors into five indicators: enterprises, total precipitation, social public vehicles, wind speed, and green coverage in the built-up area. These factors can effectively explain the PM2.5 spatially stratified heterogeneity for more than half of the three major urban agglomerations of the YREB and can be applied to the spatial analysis models as the main impact factors of PM2.5. It is found that the superposition of meteorological factors and socio-economic indicators will have a significant heterogeneity effect in the PM2.5 distribution. (4) The GWR and MGWR models were used for spatial regression. In the quantitative analysis, the MGWR model showed better fitting regression and smaller errors and was more suitable for studying spatial characteristics on a large-scale. On this basis, the MGWR model was adopted to analyze the most important factors of the three urban agglomerations, and we found that the most important driving force factors of PM2.5 in 2015–2017 differed from both spatial and temporal perspectives. As the most influential driving factor, wind speed affected the spatial distribution of PM2.5 in the three urban agglomerations along the YREB in 2018. In addition, future research will focus on eliminating the loss of sequence rules caused by the data averaging process and improving the accuracy of PM2.5 spatial modeling. In order to better control air pollution, the PM2.5 concentration prediction model is also one of the key points of future research.

## Figures and Tables

**Table 1:** Research data and uses.

Theme	Variable Name	Unit	Description	Sampling Interval
PM2.5	(1) Y: fine particulate matter	μg/m^3^	(1) Describes the main object in the study	Hour
Economics	(1) X1: Gross domestic product	100 million yuan	(1) Describes the economic development of the city	Year
	(2) X2: Proportion of secondary industry	%	(2) Describes the level of city industry	Year
	(3) X3: Enterprises	count	(3) Describes the pollution level of urban enterprises	Year
Traffic	(1) X4: Road area	10,000 sq.m	(1–2) Describes the level of urban traffic pollution	Year
	(2) X5: Social public vehicles	count		Year
City attribute	(1) X6: Population density	people/km^2^	(1) Describes the distribution of the urban population	Year
	(2) X7: Green coverage in the built-up area	%	(2) Describes the greening level of urban built-up areas	Year
Education	(1) X8: Middle school	count	(1) Describes the level of basic urban education	Year
Meteorological factors	(1) X9: Minimum temperature	°C	(1–7) Describes the weather conditions of the city	Hour
	(2) X10: Maximum temperature	°C	-	Hour
	(3) X11: Average temperature	°C	-	Hour
	(4) X12: Humidity	%	-	Hour
	(5) X13: Wind speed	m/s	-	Hour
	(6) X14: Pressure	hPa	-	Hour
	(7) X15: Total precipitation	mm	-	Hour

**Table 2:** Global Moran’s Index of the PM2.5 concentrations of YRD-UA and its significance tests.

Time	Moran’s I	Z(I)	*p*	Time	Moran’s I	Z(I)	*p*
Average	0.517	4.821	<0.01	January	0.590	5.379	<0.01
2015	0.285	2.788	<0.01	February	0.539	4.865	<0.01
2016	0.430	4.126	<0.01	March	0.505	4.707	<0.01
2017	0.573	5.116	<0.01	April	0.304	3.038	<0.01
2018	0.610	5.424	<0.01	May	0.430	3.981	<0.01
Spring	0.647	5.744	<0.01	June	0.443	4.070	<0.01
Summer	0.429	4.101	<0.01	July	0.427	3.855	<0.01
Autumn	0.363	3.391	<0.01	August	0.206	2.068	0.04
Winter	0.481	4.475	<0.01	September	0.291	2.871	<0.01
-	-	-	-	October	0.511	4.634	<0.01
-	-	-	-	November	0.545	4.986	<0.01
-	-	-	-	December	0.539	4.936	<0.01

**Table 3:** Global Moran’s Index for the PM2.5 concentrations of YMRM-UA and its significance tests.

Time	Moran’s I	Z(I)	*p*	Time	Moran’s I	Z(I)	*p*
Average	0.634	5.420	<0.01	January	0.718	6.198	<0.01
2015	0.745	6.238	<0.01	February	0.696	5.910	<0.01
2016	0.614	5.225	<0.01	March	0.722	6.147	<0.01
2017	0.335	3.073	<0.01	April	0.333	2.997	<0.01
2018	0.502	4.413	<0.01	May	0.435	3.950	<0.01
Spring	0.604	5.143	<0.01	June	0.390	3.497	<0.01
Summer	0.172	1.726	0.08	July	−0.017	0.163	0.87
Autumn	0.374	3.363	<0.01	August	0.037	0.615	0.54
Winter	0.694	6.012	<0.01	September	0.262	2.437	0.02
-	-	-	-	October	0.119	1.272	0.20
-	-	-	-	November	0.498	4.443	<0.01
-	-	-	-	December	0.626	5.438	<0.01

**Table 4:** Global Moran’s Index for the PM2.5 concentrations of CY-UA and its significance test.

Time	Moran’s I	Z(I)	*p*	Time	Moran’s I	Z(I)	*p*
Average	0.126	1.425	0.15	January	0.123	1.314	0.19
2015	−0.074	−0.081	0.94	February	0.178	1.802	0.07
2016	0.164	1.622	0.10	March	0.277	2.427	0.02
2017	0.286	2.618	<0.01	April	0.209	1.962	0.05
2018	0.010	0.552	0.58	May	0.207	2.007	0.04
Spring	0.125	1.331	0.18	June	−0.043	0.135	0.89
Summer	0.248	2.282	0.02	July	−0.196	−0.940	0.35
Autumn	−0.108	−0.323	0.75	August	−0.089	−0.188	0.85
Winter	0.149	1.583	0.11	September	−0.028	0.235	0.81
-	-	-	-	October	0.099	1.225	0.22
-	-	-	-	November	0.372	3.145	<0.01
-	-	-	-	December	0.002	0.442	0.66

**Table 5:** Variance inflation factors (VIFs) statistical table for each factor.

Variable	VIFs	State
X10: Maximum temperature	9.155588111575856	Failed
X12: Humidity	1.831256992288039	Pass
X13: Wind speed	1.7003003448074754	Pass
X14: Pressure	14.098901165524186	Failed
X15: Total precipitation	4.594658800736686	Pass

**Table 6:** Factor_detector results.

	X1	X2	X3	X4	X5	X6	X7	X8	X12	X13	X15
q statistic	0.0488	0.0388	0.1749	0.0432	0.0768	0.0243	0.0534	0.0518	0.0269	0.0618	0.1179
*p* value	0.000	0.000	0.000	0.000	0.000	0.000	0.000	0.000	0.000	0.000	0.000

**Table 7:** Ecological_detector results.

Variable	X1	X2	X3	X4	X5	X6	X7	X8	X12	X13	X15
X1	-	-	-	-	-	-	-	-	-	-	-
X2	N	-	-	-	-	-	-	-	-	-	-
X3	Y	Y	-		-	-	-	-	-	-	-
X4	N	N	Y	-	-	-	-	-	-	-	-
X5	N	N	Y	N	-	-	-	-	-	-	-
X6	N	N	Y	N	Y	-	-	-	-	-	-
X7	N	N	Y	N	N	N	-	-	-	-	-
X8	N	N	Y	N	N	N	N	-	-	-	-
X12	N	N	Y	N	Y	N	N	N	-	-	-
X13	N	N	Y	N	N	N	N	N	N	-	-
X15	Y	Y	Y	Y	N	Y	Y	Y	Y	Y	-

**Table 8:** Interaction_detector results.

Variable	X1	X2	X3	X4	X5	X6	X7	X8	X12	X13	X15
X1	0.0488	-	-	-	-	-	-	-	-	-	-
X2	0.2558	0.0389	-	-	-	-	-	-	-	-	-
X3	0.2786	0.3439	0.1749	-		-	-	--	-	-	-
X4	0.1402	0.3034	0.3380	0.0432	-	-	-	-	-	-	-
X5	0.2013	0.2778	0.3613	0.2085	0.0768	-	-	-	-	-	-
X6	0.2678	0.2221	0.3029	0.2026	0.1782	0.0243	-	-	-	-	-
X7	0.2556	0.2279	0.3265	0.3128	0.2469	0.2956	0.0534	-	-	-	-
X8	0.2945	0.2696	0.3389	0.2676	0.3106	0.2767	0.3165	0.0518	-	-	-
X12	0.2176	0.2032	0.3476	0.179	0.2471	0.2167	0.2085	0.2074	0.0269	-	-
X13	0.2654	0.2474	0.3562	0.2606	0.2556	0.2812	0.2307	0.3349	0.3046	0.0618	-
X15	0.2760	0.2907	0.3547	0.2897	0.2629	0.2650	0.3043	0.3007	0.2866	0.2974	0.1179

**Table 9:** Comparison of the GWR and MGWR parameters.

Analysis Index	2015		2016		2017		2018	
	GWR	MGWR	GWR	MGWR	GWR	MGWR	GWR	MGWR
Residual Squares	5016.893	41.043	3020.999	44.202	3765.526	51.354	3245.950	46.311
Effective Number	10.960	12.145	10.930	11.893	10.745	11.098	10.995	11.474
Sigma	9.066	0.828	7.033	0.858	7.840	0.918	7.294	0.875
AICc	534.385	196.576	498.050	201.150	513.242	209.572	503.022	203.248
R2	0.283	0.430	0.253	0.386	0.177	0.287	0.226	0.357
R2Adjusted	0.166	0.312	0.132	0.263	0.046	0.155	0.010	0.233

**Table 10:** 2015–2018 MGWR Bandwidth statistics table.

Variable	2015		2016		2017		2018	
	Bandwidth	ENP	Bandwidth	ENP	Bandwidth	ENP	Bandwidth	ENP
Intercept	43.000	2.902	43.000	2.902	43.000	2.999	43.000	3.022
X3	71.000	1.658	71.000	1.755	71.000	1.646	71.000	1.599
X5	71.000	1.846	71.000	1.911	71.000	1.761	71.000	1.793
X7	46.000	2.446	44.000	2.320	71.000	1.498	68.000	1.608
X13	62.000	1.658	71.000	1.591	65.000	1.730	65.000	1.830
X15	70.000	1.471	71.000	1.415	68.000	1.464	71.000	1.622

**Table 11:** 2015–2018, table of the total regression coefficient for each urban agglomeration.

Year	UA	X3	X5	X7	X13	X15
2015	CY-UA	0.0501	0.1838	−0.0519	0.2845	−0.2318
	YRMR-UA	0.0321	0.1153	−0.3978	0.1060	−0.3210
	YRD-UA	0.0297	0.0362	−0.2294	0.1841	−0.3227
2016	CY-UA	−0.1898	0.4302	−0.1964	0.2794	−0.0626
	YRMR-UA	−0.1097	0.2146	−0.1439	0.1891	0.0130
	YRD-UA	−0.1235	0.1102	−0.0521	0.1545	0.0048
2017	CY-UA	−0.0926	0.1156	0.0154	0.2064	−0.2524
	YRMR-UA	−0.1633	0.0668	−0.0567	0.1609	−0.0102
	YRD-UA	−0.2467	0.0171	−0.0654	0.0672	0.0147
2018	CY-UA	−0.1760	0.0942	0.1113	0.3328	−0.2483
	YRMR-UA	−0.1482	0.0803	−0.0967	0.3372	−0.1990
	YRD-UA	−0.2029	0.0219	−0.1258	0.2824	−0.2103

## Data Availability

Publicly available datasets were analyzed in this study. The PM2.5 data were collected from the China National Environmental Monitoring Station. Meteorological data were collected from the China meteorological data network and the Hui-Ju data. Data on economics, traffic, city attributes, and education were collected from the National Bureau of Statistics released by the China statistical yearbook. Population density and green coverage in the built-up area were collected from the National Bureau of Statistics released by the China city statistical yearbook. These data can be found here: http://www.cnemc.cn last accessed on 15 July 2020; http://data.cma.cn/market/index.html last accessed on 20 July 2020; http://hz.zc12369.com/home/meteorologicalData/dataDetailsThreeYear/ last accessed on 21 July 2020; http://www.stats.gov.cn/tjsj/ndsj/ last accessed on 18 July 2020; http://www.stats.gov.cn/zjtj/gjtjj/201311/t20131108_457871.html last accessed on 18 July 2020.
